# Host and brood parasite coevolutionary interactions covary with comparative patterns of the avian visual system

**DOI:** 10.1098/rsbl.2021.0309

**Published:** 2021-09-29

**Authors:** Ian J. Ausprey, Mark E. Hauber

**Affiliations:** ^1^ Florida Museum of Natural History and Department of Biology, University of Florida, Gainesville, FL 32611-7011, USA; ^2^ Department of Evolution, Ecology, and Behavior, School of Integrative Biology, University of Illinois at Urbana-Champaign, Champaign, IL, USA

**Keywords:** coevolutionary arms-race, egg mimicry, residual eye size, visual system

## Abstract

In coevolutionary arms-races, reciprocal ecological interactions and their fitness impacts shape the course of phenotypic evolution. The classic example of avian host–brood parasite interactions selects for host recognition and rejection of increasingly mimetic foreign eggs. An essential component of perceptual mimicry is that parasitic eggs escape detection by host sensory systems, yet there is no direct evidence that the avian visual system covaries with parasitic egg recognition or mimicry. Here, we used eye size measurements collected from preserved museum specimens as a metric of the avian visual system for species involved in host–brood parasite interactions. We discovered that (i) hosts had smaller eyes compared with non-hosts, (ii) parasites had larger eyes compared with hosts before but not after phylogenetic corrections, perhaps owing to the limited number of independent evolutionary origins of obligate brood parasitism, (iii) egg rejection in hosts with non-mimetic parasitic eggs positively correlated with eye size, and (iv) eye size was positively associated with increased avian-perceived host–parasite eggshell similarity. These results imply that both host-use by parasites and anti-parasitic responses by hosts covary with a metric of the visual system across relevant bird species, providing comparative evidence for coevolutionary patterns of host and brood parasite sensory systems.

## 1. Introduction

In coevolutionary interactions, reciprocally interacting lineages shape each other's ecological and physiological milieus and generate selective forces to impact the direction and pace of evolution [1]. A classic example is the relationship between avian obligate brood parasites and their hosts. In this case, host specialization by parasites may select for nesting behaviours that limit access of parasitic intruders into increasingly enclosed host nest structures [2]. Likewise, some hosts are better able to recognize and reject foreign eggs, even when the parasitic eggshells are highly mimetic in background coloration and maculation patterns [3,4].

Although frequently hypothesized in the context of avian host–parasite coevolution [5,6], direct morphological and physiological evidence is still lacking for the evolutionary impact of brood parasitism upon the sensory systems of hosts and their parasites. This is surprising because visible cues at a distance and, hence, greater visual acuity through relatively larger eyes [7] are both critical for parasites to locate host nests [8] and for hosts to recognize and prevent, dampen or eliminate parasitism (e.g. the detection of approaching adults, parasitic eggs and/or hatched chicks [9]). As the rare exception, one study examined whether a short-wavelength (SWS I) avian visual receptor was more often tuned for ultraviolet wavelengths in rejector over acceptor host species in a handful of passerine lineages but found no statistical evidence for such a predicted pattern [10]. Similarly, relative overall brain size (an indirect predictor of neural processing complexity of visual information such as the coloration of foreign eggs or the sight of well-hidden host nests) was not predictably larger in egg rejector compared with non-rejector host individuals intraspecifically [11] and was even smaller in rejector compared with acceptor hosts and in parasitic compared with non-parasitic lineages [12,13], although no study to date has assessed visual subregions in host or parasite brains. More specifically, no study has yet examined the role of eye morphology *per se* in mediating avian host–brood parasite interactions and coevolutionary paths [8].

Here, we employ a comparative approach based on published datasets regarding egg rejection rates [12] and eye size collected from preserved avian museum specimens [14]. We use absolute eye size (AES) as a proxy metric of a bird's visual system and its various sensitivities regarding pattern and luminance discrimination, based on the known positive correlations between these sensitivity metrics and eye size [15]. Additionally, we use residual eye size (RES) as an indicator of adaptations to pattern and luminance perception beyond expected body-size allometric relationships [14]. Although eye size *per se* is not a known predictor of greater colour discrimination, both the eggshell's maculation pattern [4,7] and its background colour luminance [3,16,17] are known to be mimicked by parasitic eggshells. Thus, visual acuity, which is positively related to greater AES and RES, likely affects the recognition of cues used by hosts when rejecting foreign eggs [7].

Accordingly, we assessed the following predictions of our novel Coevolved Sensory System Hypothesis as applied to eye size across a large sample of avian lineages: (i) eyes of brood parasites should be larger than those of hosts as many parasites use visual cues of host breeding activity to locate nests for future parasitism [18,19], (ii) hosts should have smaller eyes than non-hosts if parasites use potential host species with lower than expected visual sensitivities and reduced ability to discriminate among objects in the nest, including the recognition of foreign eggs [20], and eye size of hosts should positively covary with both (iii) parasite-egg rejection patterns by the same species and (iv) the extent of avian-perceivable, visible host-eggshell mimicry by parasites.

## 2. Methods

We extracted data on avian eye size, body mass, habitat, foraging behaviour and diet from a previously published dataset regarding the ecological correlates of eye size for a third of terrestrial bird diversity (*N* = 2777 species from 139 families) [14]. This yielded data for 750 host species, 42 brood parasites and 1985 non-hosts, generated without knowledge of the focal hypotheses and predictions addressed in this study. All eye measurements were originally collected by Stanley Ritland from whole eyes preserved in alcohol/formaldehyde as part of his unpublished dissertation [21]. Specifically, he carefully removed whole eyes from specimens and measured the transverse diameter (TD) and axial diameter (AD) using 0.05 mm Vernier calipers. He noted that the preservation process did not systematically alter the size or shape of specimens nor produce measurements notably different from freshly harvested eyes. We used TD because (i) Ritland noted that his measurements of TD were more accurate than AD, and (ii) a previous analysis using the same dataset produced similar results between the two metrics [14]. We defined habitat as forest specialist versus generalist/non-forest specialist using classifications published by BirdLife International [22]. For foraging behaviour, we used previously published databases to score species as either ‘myopic’ (near-sighted manoeuvres such as glean or probe) or ‘hyperopic’ (far-sighted manoeuvres such as sally or pounce) [23,24]. Foraging stratum and diet were extracted from the Elton Traits database [25]. See [14] for details on the assembly and scoring of these variables.

We sourced host status, the identities of host-specific parasites, and egg rejection rates for hosts of obligate avian brood parasites also from previously published databases [2,12,26]. This information was then annotated regarding whether the respective parasite laid an avian-perceived mimetic or non-mimetic egg as modelled by the particular host's visual system [16,27] and indicated by the respective spectral and perceptual review literature (e.g. [28,29]). Both eye size and rejection rate data were available for 33 host species of egg-mimetic parasites and 75 hosts of non-mimetic parasites. We additionally extracted previously published data on egg background colour and spot pattern mimicry for 10 host species of the obligate brood parasitic Common cuckoo (*Cuculus canorus*) [3,4], for which we also had eye-size measurements.

## 3. Analyses

To correct for body-size allometry, we calculated RES by extracting the residuals from a phylogenetic regression of log(TD) on log(mass) using a model of evolution incorporating Pagel's lambda. We ran the analysis across 100 hypothesized trees from a previously published phylogeny [30] and extracted residuals from the tree that produced the median slope coefficient (see [14] for details).

We first used phylogenetic linear regression to compare AES eye size and RES among hosts, brood parasites, and all other species while controlling for known correlations with habitat, foraging manoeuvre, foraging stratum and diet [14]. We ran the analysis across 100 hypothesized trees from a previously published phylogeny [30] and extracted the median estimated marginal means of coefficients for inference using *p*-values calculated from pairwise contrasts corrected for multiple comparisons to assess significance [31]. We repeated the analysis using ordinary least-squares regression, because nest parasitism is conserved within few families [2], and high phylogenetic signal may obscure the ecological relationship between eye size and nest parasitism status.

Second, we used phylogenetic linear regression to determine if eye size interacted with the mimetic status of parasitic eggs to predict host rejection rates while controlling for host habitat associations, foraging behaviour and diet. Third, we examined the degree to which eye size predicted background eggshell colour overlap and shared maculation traits for 10 species of passerines parasitized by the Common cuckoo while controlling for host rejection rate [3,4]. Here we used ordinary least-squares regression (as *N* < 20 species). All analyses were run in R 4.0 using the ‘emmeans’, ‘emmeans’, ‘nlme’, ‘phylolm’ and ‘visreg’ packages [31–34].

## 3. Results

**Figure 1.**
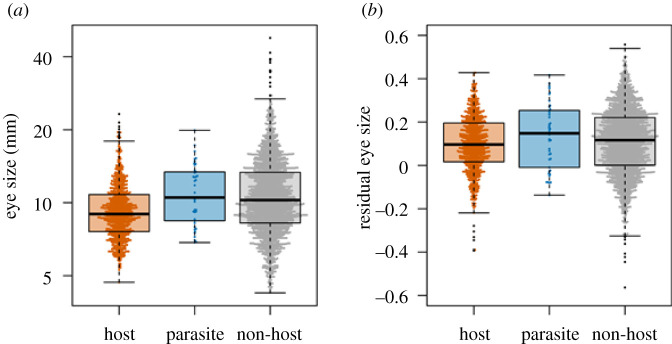
(*a*) Absolute and (*b*) residual eye size for hosts (*N* = 750), brood parasites (*N* = 42) and non-hosts (*N* = 1985).

On average, obligate avian brood parasites had larger eyes compared with hosts for the non-phylogenetic model (*β* = 0.24, *p* < 0.001), and 84% of parasite–host interactions involved parasites with larger eyes (figure 1 and electronic supplementary material, figure S1). Eye size did not differ significantly for the phylogenetic regression model (*β* = 0.002, *p* = 0.99), likely because brood parasitism has independently evolved only within a handful of avian families (*N* = 7 origins, e.g. [2]). Hosts had significantly smaller eyes than non-hosts for both non-phylogenetic (*β* = −0.11, *p* < 0.001) and phylogenetic models (*β* = −0.02, *p* < 0.02). Results were statistically similar for RES (table 1).

**Table 1.** Pairwise contrasts for (*a*) absolute and (*b*) residual eye size among hosts (*N* = 750), brood parasites (*N* = 42), and non-hosts (*N* = 1985).

	non-phylogenetic				phylogenetic			
	coef.	s.e.	*t*-stat	*p*-value	coef.	s.e.	*t*-stat	*p*-value
(*a*)								
host versus parasite	−0.237	0.042	−5.58	<0.001	−0.002	0.052	−0.04	0.993
host versus non-host	−0.111	0.012	−9.43	<0.001	−0.016	0.006	−2.62	0.024
non-host versus parasite	0.125	0.042	2.99	0.008	−0.015	0.052	−0.29	0.954
(*b*)								
host versus parasite	−0.077	0.019	−4.06	<0.001	0.011	0.029	0.40	0.917
host versus non-host	−0.001	0.005	−0.14	0.989	−0.012	0.004	−2.85	0.012
non-host versus parasite	0.076	0.019	4.07	<0.001	−0.023	0.029	−0.81	0.695

**Figure 2.**
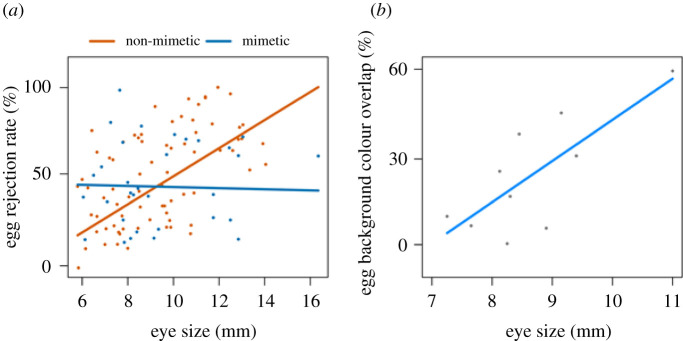
Partial residual plots from linear regressions of eye size on (*a*) rejection rate for hosts of brood parasites with non-mimetic (*N* = 75) and mimetic (*N* = 33) eggs, and (*b*) background colour overlap of eggs between 10 hosts and their common parasite, the Common cuckoo.

Hosts with larger eyes rejected eggs of non-mimetic parasites at an increased rate while controlling for phylogeny (*β* = 9.9, 95% CI = 5.7–14.2) (figure 2*a*). Rejection rates for eggs of mimetic parasites, however, were not related to eye size (*β* = −0.7, 95% CI = −6.2–4.8). Results for RES were similar for non-mimetic (*β* = 60.5, 95% CI = 0.5–120.9) and mimetic parasites' eggs (*β* = −9.0, 95% CI = −98.9–80.2).

The handful of hosts whose published egg colour overlapped more with those of their brood parasite (*N* = 10 Common cuckoo host species) [3,4] had larger eyes while controlling for rejection rate (*β* = 10.1, *p* = 0.02) (figure 2*b*), increasing the *R*^2^ value for the previously published univariate rejection rate model from 0.47 to 0.77 [3]. Results were similar for RES (*β* = 135.7, *p* = 0.03, *R*^2^ = 0.74). There was no relationship between the published host–parasite eggshell maculation pattern overlap and host eye size (absolute: *β* = 5.7, *p* = 0.43; residual: *β* = 110.2, *p* = 0.25).

## 4. Discussion

Our comparative analyses generated directional, albeit correlational, support for three predictions regarding eye size correlates of host–brood parasite interactions across the avian order. First, eyes of parasites were found to be larger than those of hosts (figure 1 and table 1). This pattern was detected only for the non-phylogenetic model, implying that phylogenetic inertia outweighed the effects of ecological differences in the phylogenetic models. This is expected since avian brood parasitism has only evolved at seven independent origins of avian diversity [2], linking most of the approximately 100 avian obligate brood parasite species through often shared phylogenetic histories. Although this result may have arisen because brood parasites, in general, are larger in body size than hosts [10] and eye size is positively related to body dimensions [12], results were similar when using RES.

Second, hosts had smaller eyes than non-hosts, suggesting that brood parasites target potential hosts with reduced visual sensitivities that are less able to recognize novel/foreign objects in the nest. This conclusion held true in the phylogenetic analyses of both AES and, critically, RES, implying that parasites are not simply selecting hosts of smaller body size [35]. Nonetheless, there was substantial overlap between our distinct categories of avian species, implying that detecting these patterns among diverse groups of avian lineages with varied sensory ecologies [14] can generate low biological separation, even when statistically significant.

Third, we demonstrated that eye size positively covaries with egg rejection patterns across some hosts of parasites; this pattern held true again across both AES and RES but did so only for hosts of non-mimetic egg-laying parasites. This is expected because in non-coevolved host–parasite pairs (such as those with non-mimetic parasite eggs), broad metrics of eye-sight acuity (such as residual eye size) should positively predict egg rejection patterns, whereas in coevolved host–parasite pairs (such as those with mimetic parasite eggs), additional cognitive decision rules and other socio-ecological adaptations (e.g. prior experience [36]) are likely to determine egg recognition and rejection patterns instead.

Finally, in a subset of host species parasitized by the mimetic egg-laying Common cuckoo host races, the extent of avian-perceivable mimicry was positively correlated with both AES and RES when controlling for egg rejection rates, implying that coevolutionary histories yielding increasingly mimetic parasitic egg appearances are predictably and positively associated with eye size metrics.

These results support the idea that hosts experience a significant optical disadvantage in defending nests against parasites, given that the vast majority of host–parasite interactions involved parasites with predictably superior visual systems. Larger eyes have more retinal ganglia cells, collect more light and are thought to expand the perceptual range by improving visual acuity and sensitivity to contrast [37–39]. Despite the disadvantage of having smaller eyes than parasites on average, hosts with larger eyes, and presumably enhanced visual acuity [15] were more likely to reject non-mimetic parasitic eggs, potentially due to increased discrimination ability between distinctly divergent egg shell background coloration, chromaticity and maculation [7]. In particular, the positive correlation in background eggshell colour overlap and eye size suggests an arms-race between the evolution of mimetic parasitic eggs and host visual ability. Although eye size is not an indicator of microanatomical structures that interpret colour cues [40], the identification of both eggshell achromatic (luminance) cues and the colour and pattern of maculations is known to be involved in cueing egg rejection and is presumably improved by increased visual acuity [7,15].

Overall, we demonstrate predicted statistical linkages between hosts, parasites and gross anatomical metrics of their primary visual organ, the avian eye, across a large diversity of bird species. Critically, both absolute and residual eye metrics showed covariation with host–parasite coevolutionary status across our analyses. This research highlights the need for future detailed visual system and neural pathway analyses (e.g. [41]) of hosts of both those parasitic lineages that lay mimetic eggs and those that lay non-mimetic eggs, so that we can directly compare the sensory systems of hosts as a function of their coevolutionary history with obligate avian brood parasites.

## Data accessibility

All data and scripts are available on Dryad: https://doi.org/10.5061/dryad.vmcvdncsp.
